# It takes acid, rather than ice, to freeze glucose

**DOI:** 10.1038/srep08875

**Published:** 2015-03-09

**Authors:** S. A. A. van den Berg, M. H. M. Thelen, L. P. W. Salden, S. W. van Thiel, K. J. M. Boonen

**Affiliations:** 1 Dept. of Clinical Chemistry and Hematology, Amphia Hospital, Breda, The Netherlands; 2 Dept. of Internal Medicine, Amphia Hospital, Breda, The Netherlands

## Abstract

Plasma glucose levels provide the cornerstone of diabetes evaluation. Unfortunately, glucose levels drop *in vitro* due to glycolysis. Guidelines provide suitable conditions which minimize glycolysis, such as immediate centrifugation or the use of ice/water slurry storage containers. For obvious practical reasons, most laboratories use blood collection tubes containing glycolysis inhibitors. We describe the effect of a variety of commonly used blood collection tubes on *in vitro* stability of glucose. Furthermore, we looked at the validity of the assumption that glycolytic activity is minimal when blood is kept in an ice/water slurry. Sodium fluoride alone does not reduce *in vitro* glycolysis in the first 120 minutes after phlebotomy. Addition of citrate almost completely prevented *in vitro* glycolysis, but showed a positive bias (0.2 mmol/l) compared to control. This is partly due to a small drop in glucose level in control blood, drawn according to the current guidelines. This drop occurs within 15 minutes, in which glycolysis has been described to be minimal and acceptable. NaF-EDTA-citrate based test tubes provide the best pre-analytical condition available. Furthermore, glucose levels are not stable in heparinized blood placed in an ice/water slurry. We strongly advise the use of NaF-EDTA-citrate based test tubes in diabetes research.

Although under debate^1^,^2^, plasma glucose concentration is still widely used in the diagnostic workup of type II diabetes mellitus (DM) as well as gestational diabetes. It is well known that (pre)analytical variables such as the choice of phlebotomy material and post-phlebotomy turn-around-time (TAT) affects *in vitro* glucose stability and thus, concentration^3^. *In vitro*, glucose levels may drop as much as 7% per hour (±0.6 mmol/l/h) due to ongoing glycolysis^4^,^5^. Furthermore, secondary factors such as leukocyte count^6^ and ambient temperature may even double *in vitro* glycolytic rate. In patients with chronic leukemia, glucose concentration might drop 10% to 20%, depending on the white cell count^6^. Given the stringent cut off levels set for the diagnosis of DM, a false low glucose determination may possibly result in clinical misdiagnosis and subsequent harm to the patient^7^ and increased health cost.

To guarantee an accurate glucose determination, in particular for outpatient situations, it is common practice to use collection tubes that contain anti-glycolytic agents^8^. The mode of action of the most commonly used anti-glycolytic agent, sodium fluoride (NaF), is based on the inhibition of enolase activity^9^. Although inhibition of enolase activity stabilizes the glucose concentration in the long term, it does not prevent a drop during the first hours after phlebotomy^5^. Using NaF alone as a glycolytic inhibitor is therefore considered insufficient. The initial drop in glucose concentration can be prevented by rapid centrifugation after phlebotomy. In addition, storage of the collection tube in an ice/water slurry or acidification of the collected blood are considered good alternatives^3^.

Recently, we published the results of a survey on common laboratory procedures in the context of glucose analysis, which was held among Dutch clinical chemistry laboratories^10^. Surprisingly, less than 5% of the laboratories used one of the recommended protocols^3^. Even though TAT was typically longer than 60 minutes, most laboratories undertook no action, other than to use NaF based phlebotomy material, to prevent *in vitro* glycolysis. None of the laboratories used an ice/water slurry and only 2 out of 25 laboratories used phlebotomy material that contained an acidic additive.

There have been a large number of reports where citrated additives have indeed been shown to prevent glycolysis and stabilize *in vitro* glucose concentration (reviewed in^11^). Most reports compare long term (≥4 hours) stability of glucose concentrations in citrated samples versus non-citrated samples. However, only a few studies have studied time dependent changes that occur during the first 2 hours after phlebotomy, a situation more commonly encountered in a hospital situation. Furthermore, to our knowledge, only one of the published studies focused on within subject changes; a study design that requires paired sampling of control material^12^.

Here, we describe a comprehensive and detailed study in which we studied the effect of commonly used types of phlebotomy material, turn-around-time and the interaction between these two parameters on *in vitro* glucose concentration. We focused on common laboratory conditions and assessed the validity of the assumption that glycolytic activity is minimal when phlebotomy material is kept in an ice/water slurry prior to centrifugation and analysis.

## Methods

### Subjects

The study described here was conducted according to the principles of the Declaration of Helsinki, adapted in 2013 (Fortaleza, Brazil) and in accordance with the Dutch Medical Research Involving Human Subjects Act (WMO) and was registered as trial number NL46462.015.13.

In part 1, blood was drawn from apparently healthy volunteers (volunteer hospital staff, n = 88). 4 equal groups of 22 test subjects were created by stratification by age and sex. In total, 5 test tubes were drawn from each volunteer. From each individual, a lithium-heparin tube (throughout the manuscript denoted as “control”) was collected (BD Vacutainer, 367374), which was placed in an ice/water slurry for a maximum of 15 minutes prior to centrifugation (5 minutes, 2800 g). This design enabled individual, pairwise comparison of glucose concentration to that individuals own control tube. Subsequently, in each volunteer 4 additional test tubes were drawn of 1 specific tube type. In group 1; 4 additional lithium-heparin tubes were drawn. In group 2; 4 tubes containing NaF-EDTA-citrate (Terumo Venosafe VF-052SFC) were drawn. In group 3; 4 tubes containing NaF-oxalate (BD Vacutainer 368921) and in group 4; 4 tubes containing NaF-EDTA (BD Vacutainer 368521). Similar to the control tube, 1 test tube was placed in an ice/water slurry and centrifuged within 15 minutes after phlebotomy (time point = 0 minutes). The other test tubes were stored at room temperature, and centrifuged at set time points (30, 60 and 120 minutes) after phlebotomy. Subsequently, plasma was collected and stored at −80° until further analysis.

In part 2, blood was drawn from apparently healthy volunteers (n = 7) in a lithium-heparin tube (BD Vacutainer, 367374), which was placed in an ice/water slurry. At set time points (0, 3, 6, 9, 12, 15 minutes) a small sample was withdrawn from the test tube, transferred into pre-chilled micro tubes and centrifuged immediately in a pre-chilled centrifuge (4°, 3 minutes, 18.000 g). After centrifugation plasma was collected and stored on ice prior to analysis. All analyses were performed in the same run of the same analyzer which met internal and external quality control criteria, directly after collection of the 15 minute timepoint.

In separate lithium-heparin tubes, core temperature was measured at set time points (0, 3, 6, 9, 12, 15 minutes) after collection in tubes placed at room temperature 22° (n = 3) or in an ice/water slurry (n = 3).

### Test characteristics

Glucose concentration measurements were performed at the laboratory of clinical chemistry and hematology, Amphia Hospital on a Roche Cobas C501 analyzer (GLUC3 application, Roche, Mannheim, Germany). Day-to-day analytical imprecision (standard deviation) was 0.08 mmol/L at glucose concentration 4.4 mmol/l.

### Statistics

Glucose concentration in the control tubes of each experimental group was compared by one-way ANOVA, followed by a Bonferroni post-hoc test. The difference in the drop in glucose concentration in the various test tubes when compared to the drop in lithium-heparin tubes was determined by two-way ANOVA, respectively, followed by a Bonferroni post-hoc test. Difference in free hemoglobin and icteric index compared to control was determined by two-way ANOVA, respectively, followed by a Bonferroni post-hoc test. Within test group differences were assessed by paired T-Test. For all comparisons, threshold for statistical significance was set at 5%. Throughout the manuscript, data is represented as mean ± standard deviation.

## Results

### NaF-EDTA-citrate prevents in vitro glycolysis

The average glucose concentration in the control tubes was similar between the 4 experimental groups, lithium-heparin, NaF-EDTA and NaF-oxalate and NaF-EDTA-citrate (5.6 ± 0.7; 5.7 ± 0.9; 5.8 ± 1.5 and 5.8 ± 1.0 mmol/l), respectively.

**Figure 1:**
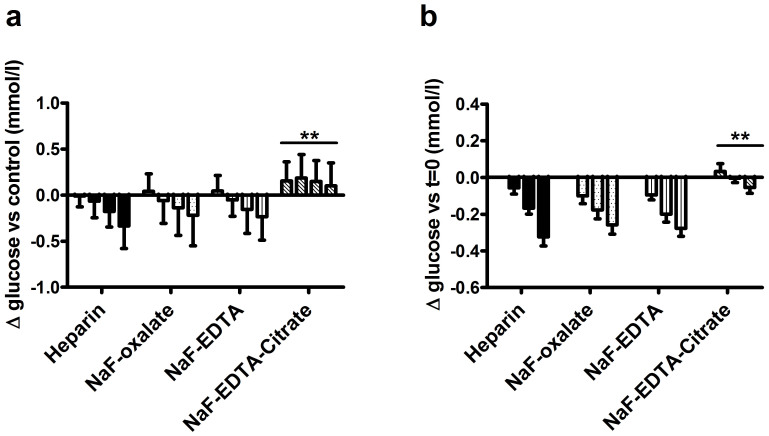
Time and tube type dependent changes of glucose concentration. (A): Glucose concentration difference compared to control (lithium-heparin, kept on ice, measured at t = 0). Bars represent the different time points, from left to right: 0, 30, 60, 120 minutes. (B): Time dependent changes in glucose concentrations compared to the concentration measured in the same tube type at t = 0. Bars represent the different time points, from left to right: 30, 60 and 120 minutes. Bars represent mean plus SD, **p < 0.01 for tube type/time interaction by two-way ANOVA, when compared to lithium-heparin tubes.

Compared to the drop in glucose concentration in the lithium-heparin tubes, glucose concentration dropped at a similar speed in NaF-EDTA and NaF-oxalate test tubes (Figure 1a). The drop in glucose concentration was significantly less in the NaF-EDTA-citrate test group when compared to the lithium-heparin group (*p* < 0.01 and *p* < 0.001 at time points 60 and 120 minutes respectively).

Interestingly, when compared to the within test group control tubes, the glucose concentration at time point 0 was higher in all NaF based tube types. This difference was most predominant, and highly significant, in the NaF-EDTA-citrate test group (*p* < 0.01). Within the same tube type, however, glucose levels were stable in NaF-EDTA-citrate tubes, but not other tube types (*p* < 0.01, Figure 1b).

Together, these data show that glucose concentration drops with similar speed in lithium-heparin, NaF-EDTA and NaF-oxalate test tubes, but do not drop in NaF-EDTA-citrate test tubes. In addition, a positive bias in glucose concentration was found in NaF-EDTA-citrate test tubes when compared to control test tubes.

### An Ice/water slurry does not reliably prevent glycolysis in lithium-heparin tubes

**Figure 2:**
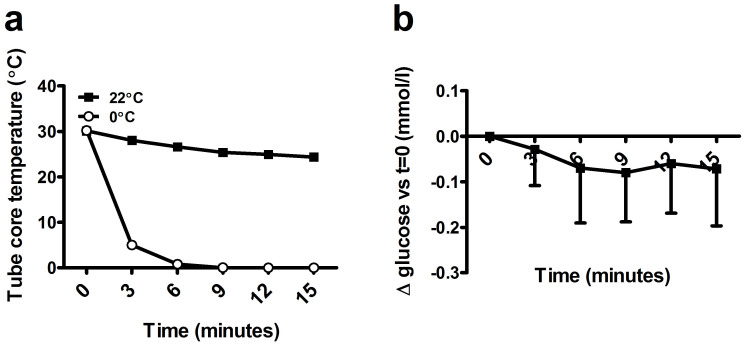
Time and temperature dependent changes of glucose concentration. (A): Time dependent change in core temperature and (B): changes in glucose concentration in heparinized blood placed in an ice/water slurry. Lines represent mean plus SD.

Given the stability of the glucose concentration in the NaF-EDTA-citrate test tubes, we hypothesized that glucose concentration might have dropped in the control tubes, even though the tubes were stored only shortly and in an ice/water slurry. A possible reason could be the time needed to cool the blood in the test tube, due to the composition of the test tube wall and the blood volume. We measured core tube temperature in 3 test tubes and found that, in ice water, core temperature dropped to approximately 5° within 3 minutes (Figure 2a). We collected heparinized blood in 7 volunteers, which was immediately placed in an ice slurry after phlebotomy. Glucose concentration dropped in 5 of the cases and although the average drop was limited, it ranged from −0.07 mmol/l to −0.29 mmol/l (Figure 2b).

Together, these data show that the drop in glucose concentration is low, but not completely prevented, in heparinized blood when the tube is placed in an ice/water slurry and that there is large variation between subjects.

The bias in glucose concentration in NaF-EDTA-citrate test tubes, when compared to immediately cooled and centrifuged control tubes, was only partially explained by the drop in glucose concentration in the control tube. One other possibility could be the relative displacement of water from the plasma to the intracellular space, due to a difference in osmolality. We measured osmolalitiy in control and NaF-EDTA-citrate plasma. Osmolality was significantly higher in NaF-EDTA-citrate plasma when compared to control plasma (322 ± 9 mOsmol/kg versus 291 ± 8 mOsmol/kg, *p* < 0.01, respectively). These data suggest that a fluid shift is unlikely to cause the higher glucose levels found in NaF-EDTA-citrate plasma, since that would result in an opposite effect on plasma glucose concentration.

### NaF-EDTA-citrate induces mild hemolysis

**Figure 3:**
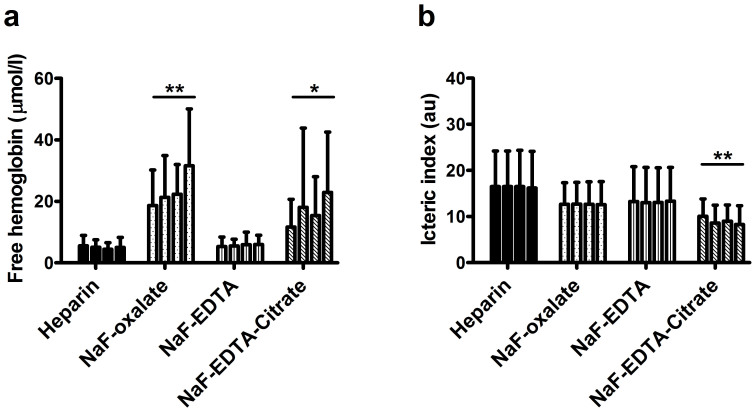
Time and tube type dependent free hemoglobin concentration and icteric index. Free hemoglobin concentration and icteric index at 0, 30, 60 and 120 minute time points, depicted per tube type, time points from left to right. Bars represent the different time points, from left to right: 0, 30, 60, 120 minutes. Bars represent mean plus SD, *p < 0.05 and **p < 0.01 for tube type/time interaction by two-way ANOVA, when compared to lithium-heparin tubes.

A commonly encountered problem with phlebotomy material with a granulated rather than sprayed on additive is an increased number of hemolytic samples. We studied free hemoglobin concentration in all test tubes. In the control tubes, free hemoglobin content was similar between groups and did not exceed 15 *μ*mol/l. Similar results were found for the icteric and lipemic indices, which were similar in control tubes of all test groups (data not shown). In NaF-EDTA test tubes, hemolytic index was similar to control tubes. In NaF-oxalate, the average hemolytic indices were significantly higher when compared to control tubes and increased with time (*p* < 0.01, Figure 3a). In NaF-EDTA-citrate tubes the average hemolytic indices were comparable to control at time point 0, but were also significantly higher when compared to control tubes and increased with time at all other time points (*p* < 0.05, Figure 3a). The 95th percentile of free hemoglobin concentration was; lithium-heparin 11, NaF-EDTA 12, NaF-oxalate 54 and NaF-EDTA-citrate 60 *μ*mol/l, when determined over all test tubes, respectively. Lipemic indices did not differ between tube types, at any of the time points (data not shown). The icteric index was stable over time within a single test group, was lower in all NaF containing tubes, but was only significantly lower in NaF-EDTA-citrate containing material (*p* < 0.01, Figure 3b). These data show that hemolytic index is higher and increases in time in NaF-oxalate and NaF-EDTA-citrate test tubes. Lipemic and icteric indices were stable in time in NaF based tubes, and either lower or comparable to indices found in lithium-heparin test tubes.

## Discussion

Here, we confirm that sodium fluoride (NaF) addition to phlebotomy material does not reduce *in vitro* glycolysis. Addition of citrate almost completely prevented *in vitro* glycolysis, but showed a positive bias in glucose concentration when compared to plasma collected from heparinized blood that is stored in an ice/water slurry. However, we also showed that this bias is probably due to a small but significant decrease in glucose concentration in plasma collected from heparinized blood that was stored in an ice/water slurry.

Glucose levels dropped similarly in all tube types, except for citrated blood. However, even though the average drop was only 0.33 mmol/L it is important to keep in mind that there are large inter-individual differences. For example, the maximum decline in glucose concentration after two hours was 0.9 mmol/L in heparinized blood, and 0.7 mmol/L in NaF-EDTA and NaF-oxalate blood. This variation in reduction in glucose concentration could be due to secondary factors such as leukocyte count^6^ and ambient temperature, which may double *in vitro* glycolytic rate.

Granulated additives, such as present in the NaF-EDTA-citrate tube used in our study, are notorious for inducing hemolysis^8^. To a large extent, this may be due to improper mixing by phlebotomists, thereby exposing a small pool of blood to high concentrations of additive. Here, we tested hemolysis as the amount of free hemoglobin detected in plasma. As was expected, hemolytic index was higher in NaF-oxalate and NaF-EDTA-citrate test tubes. Approximately 95% of samples had a free hemoglobin content below 60 *μ*mol/l, an arbitrary threshold above which a number of tests on the Roche C501 module suffer from interference. In addition, as can be seen from Figure 3, the number of samples with a high hemolytic rate increased over time, suggesting that this was due (at least in part) to an intrinsic property of the additive. In the context of glucose measurements, especially performed on analyzers employing hexokinase based tests, free hemoglobin does not interfere unless in very large amounts (≥1000 *μ*mol/l for the GLUC3 application on Roche Cobas C501)^13^. Therefore, it is unlikely that a routine sample in an NaF-EDTA-citrate test tube designated for glucose measurement will be deemed unsuitable.

The guideline that has been published concerning specimen handling and laboratory analysis of glucose states that, in addition to immediate centrifugation, glycolysis can be minimized by addition of a glycolysis inhibitor or storing the sampled material in an ice/water slurry for a maximum of 30 minutes^3^. Here, we show that even though glycolytic rate is indeed slower in material placed in an ice/water slurry, inter-individual glycolytic rates vary enormously and may even be as high as in material that is stored at room temperature. This is in line with previously published data^14^,^15^. Interestingly, our data show that the drop in glucose concentration was highest during the first 15 minutes after phlebotomy, even though the core temperature of the tube was already near 0°C. This drop might explain part of the bias found in the NaF-citrate test tube, when compared to the within group control. In addition to a drop in glucose concentration in the control tubes, a relative shift in fluid from the plasma to the intracellular compartment might explain part of the bias. We found a higher osmolality in the NaF-EDTA-citrate plasma, making a bias due to fluid shifting unlikely.

From our data, it seems that a storage period of 30 minutes in an ice/water slurry might be considered too long. With this respect, the guideline cites 2 sources. Of these sources, one is a relatively recent study^12^. However, this study did not address the time dynamic changes in glucose concentration of material that was stored in an ice/water slurry in comparison to a control sample. The second source, the 2006 WHO guideline “Definition and diagnosis of diabetes mellitus and intermediate hyperglycemia: report of a WHO/IDF consultation”, does not contain data *per se*. When tracing back, the original paper ultimately describes the stability of blood samples from adults and neonates when stored on ice. This paper indeed shows that the average drop in glucose is minimal during the first 30 minutes. However, the blood used in the study was collected in a heparinized syringe, subsequently expelled into plain 1 milliliter tubes^14^. This protocol may not reflect the current state-of-art in vacuum tube based blood sampling. More importantly, this study also shows that the variation in reduction of glycolysis is large and may be 0 in some subjects. Therefore, we question the validity of protocols that include cooling blood in ice/water for prolonged periods of time in the context of the workup for diabetes.

Here, we show that NaF-EDTA-citrate based test tubes provide the best pre-analytical condition currently available for glucose measurement. Furthermore, we show that glucose concentration is not stable in test tubes placed in an ice/water slurry.

Our data challenge all studies and recommendations that were based on measurements with delayed analysis. One might speculate that diabetes diagnosis plasma glucose cut offs would rise when based on measurements using NaF-EDTA-citrate test tubes. We therefore strongly advise that NaF-EDTA-citrate based test tubes are used in future diabetes research.

## Author Contributions

S.A.A. vdB. wrote the main manuscript, designed and executed the study. L.P.W.S. co-executed the study. M.H.M.T., S.W. vT. and K.J.M.B. co-designed the study and revised the manuscript.
